# EBP1 protein modulates the expression of human MHC class II molecules in non-hematopoietic cancer cells

**DOI:** 10.3892/ijo.2015.3051

**Published:** 2015-06-16

**Authors:** LAURA PISAPIA, PASQUALE BARBA, ANGELA CORTESE, VALERIA CICATIELLO, FRANCO MORELI, GIOVANNA DEL POZZO

**Affiliations:** Institute of Genetics and Biophysics ‘Adriano Buzzati Traverso’-CNR, 80131 Naples, Italy

**Keywords:** MHC regulation, proliferation, RNA binding protein, antigen presentation, tumor immune response

## Abstract

Many solid tumours including melanoma, glioblastoma, and breast carcinomas express MHC class II molecules (MHC II). The surface expression of these molecules confers to non-hematopoietic tumour cells the role of non-professional antigen presenting cells and the ability to potentially stimulate tumour-specific CD4^+^ T cell response. We studied EBP1, an ErbB3 binding protein, and the effects of p48 and p42 isoforms on the MHC II expression in U87 glioblastoma, M14 melanoma and MCF7 mammary carcinoma cell lines. We found that overexpression of p48 increases MHC II transcription in U87 and M14, through upregulation of CIITA transactivator and STAT1 phosphorylation. In addition, p48 protein influences MHC II expression by increasing mRNA stability. In melanoma and glioblastoma cell lines, p48 isoform functions as oncogene promoting tumour growth, while p42 isoform, that does not affect MHC II expression, acts as a tumour suppressor by blocking cell growth and inducing apoptosis. In contrast, p48 seems to act as tumour suppressor in breast carcinoma inhibiting proliferation, favouring apoptosis, and inducing a slight increase of MHC II expression similar to p42. Our data highlight the tissue specificity function of EBP1 isoforms and demonstrate that only the oncogene p48 activates MHC II expression in human solid tumours, via STAT1 phosphorylation, in order to affect tumour progression by triggering specific immune response.

## Introduction

In addition to the recognized function of cytotoxic T lymphocytes (CTLs) in immune response to cancer, it is now clear that CD4^+^ T helper lymphocytes can also participate in the generation of antitumour immunity. Tumour-infiltrating CD4^+^ T cells recognize antigens presented through MHC II, by conventional antigen-presenting cells (dendritic cells, B lymphocytes and macrophages), able to capture, process and present tumour antigens derived by cancer cells. Several published evidences demonstrated that human solid cancers of non-haematopoietic origin might express MHC II molecules and stimulate tumour antigen-specific CD4^+^ T cells that display direct cytotoxicity toward tumour cells expressing MHC II (1–3).

The MHC II expression is regulated at transcriptional level by a multi-protein enhanceosome complex that binds the W-X-Y region of MHC II promoters, stabilized by the class II transactivator CIITA (4). CIITA exhibits a cell-type constitutive or IFN-γ inducible expression profile. This cytokine activates the receptor-associated protein tyrosine kinases Janus kinase 1 (JAK1) and 2 (JAK2) and leads to the phosphorylation of signal transducer activator of transcription 1 (STAT1) that dimerizes or forms heterodimers with STAT3, translocates to the nucleus and activates its target promoters. p-STAT1 increases chromatin accessibility, through histone acetylation of CIITA promoter and induces MHC II expression (5,6).

Moreover, a post-transcriptional level of MHC II regulation has been recently characterized through the discovery of the ‘MHC II operon’ (7), a functional unit that guarantees the coordinated transcription of alpha and beta mRNAs, and the correct processing and balanced surface expression for both HLA-DR and HLA-DQ isotypes. Specifically, the MHC II operon is a ribonucleoprotein (RNP) complex in which 5′UTR and 3′UTR of MHC II mRNAs are the binding sites of a protein complex in which the RNA binding protein EBP1 (ErbB3 binding protein) has been identified (8). Several studies showed that p48, the longer isoform of EBP1, is ubiquitously expressed, both in malignant and non-malignant cells (9). Overexpression of EBP1 p48 in ErbB2/3 positive breast cancer cells and in prostate cancer cells inhibits cell growth and results in a more differentiated phenotype (10–13). Following heregulin treatment, EBP1 translocates in the nucleus where it inhibits the transcription of E2F-regulated promoters, among which E2F1 transcription factor, D1 and E cyclins (14) and androgen receptor (AR) (15). Many studies have demonstrated the suppressor role of p48 *in vivo*, as it inhibits the growth and invasion of adenoid cystic carcinoma (16) and oral squamous cell carcinoma (17). The downregulation of the longer isoform of EBP1 is also associated with hormone resistance in prostate cancer tissues (12), while in human bladder cancer tissues (18) and hepatocellular carcinoma (19) the decreased expression was associated with advanced pathologic stage and poor prognosis.

Other studies, performed in nerve growth factor (NGF)-treated PC12 cells, focused on the expression of the two isoforms of EBP1 and assigned them a different function: p48, localized in both cytoplasm and nucleus, blocks cell differentiation and promotes proliferation and survival, while the shorter form p42, localized only in the cytoplasm, suppresses cell proliferation and enhances differentiation (20). In human glioblastoma, p48 isoform is highly expressed revealing an oncogene function and a correlation with poor clinical outcome, while it facilitates tumorigenesis and enhances tumour growth in mouse xenograft models (21).

Concerning the function of RNA binding protein, it has been demonstrated that EBP1 influences the stability of *bcl2 (*22), promotes decay of AR mRNAs and inhibits its translation (23,24). Moreover, EBP1 is found within pre-ribosomal ribonucleoprotein complexes where it is involved in ribosome assembly and regulation of intermediate and late steps of rRNA processing (25,26). In the present study, we aimed to unravel the transcriptional and post-transcriptional mechanisms used by EBP1 to modulate MHC II expression in non-hematopoietic cancers. We hypothesize that the oncogene/suppressor role of p42 and p48 EBP1 isoforms, in different cells type, influences the variable MHC II expression by solid tumours, necessary to stimulate a tumor-specific immune-response of CD4^+^ T cells.

## Materials and methods

### Cell lines, reagents and transfection

M14 human melanoma and U87 human glioblastoma cells were cultured in RPMI-1640 medium, while MCF7 human breast-cancer cell line was cultured in Dulbecco’s modified Eagle’s medium (DMEM), supplemented with 10% fetal calf serum (FCS). The authenticity of cell lines, was ensured by checking HLA class II genomic asset; in particular, we carried out HLA-DRB1 and HLA-DQA1 genotypes by PCR using the AllSet Gold SSP HLA-DR Low-Resolution kit, and DQA1 SSP UniTray kit, all purchased by Invitrogen (Life Technologies Corp. Rome, Italy). The cDNA encoding p48 isoform of EBP1 protein (10) was cloned in the CMV10-3xFLAG vector (FLAG-p48). The cDNA encoding p42 isoform of EBP1 (FLAG-p42) was amplified using p48 cDNAs template, and using a forward primer starting at the third ATG (ATGATTATGGAAGAAACAGG GAAA) and a reverse primer at the transcription STOP coding sequence (TCAGTCCCCAGCTTCATTTTCT). M14, U87 and MCF7 cell lines were transfected with FLAG-p48 and FLAG-p42 constructs and several clones, analysed by FLAG expression and cells growth rate assessment, showed a comparable phenotype. M14-p48, M14-p42, U87-p48, U87-p42, MCF7-p48 and MCF7-p42 single clones were selected for the experiments described below.

The induction of MHC II expression in MCF7 was obtained by adding 500 U/ml of recombinant IFNγ (PeproTech EC Ltd., London, UK) for 48 h; STAT1 protein phosphorylation was observed after 15 min of IFNγ treatment. To measure the mRNA half-life, actinomycin D (Sigma-Aldrich), 10 μg/ml, was added to cell cultures for 2, 4 and 6 h.

### MHC II phenotype, proliferation, cell cycle and apoptosis analysis by flow cytometry

Determination of cell surface expression of MHC II antigens was performed using FACSCanto II and DIVA software (BD Biosciences, Franklin Lakes, NJ, USA). FITC mouse anti-human HLA-DR and the appropriate isotype control were purchased from BD Biosciences. For the growth rate measurements, 150,000 cells/well were plated and counted every 24 h for 4 days in triplicate.

To perform cell cycle, dynamic proliferation analysis and apoptosis, the cells were synchronized in medium deprived of serum for 42 h.

To analyse the progression of the cell cycle, the synchronized cells were released into the growth media containing 10% FBS for 8 h and then harvested, fixed and permeabilized with 1 ml of cold 70% ethanol. They were finally stained with propidium iodide 50 μg/ml, in the presence of 100 μg/ml RNase A (Serva). The distribution in cell cycle phases were analysed by FACSCanto II (BD Biosciences).

To evaluate the dynamic proliferation, synchronized cells were restarted into the growth media containing 10% FBS in the presence of EdU (5-ethynyl-2-deoxyuridine) for 24 h. The detection of EdU was performed by Click-iT EdU flow cytometry assay kit (Molecular Probes, Eugene, OR, USA). Apoptotic cells, after 24 h into growth media containing 10% FBS, were identified by double staining with Annexin V FITC kit (Miltenyi Biotec GmbH, Bergisch Gladbach, Germany), according to the manufacturer’s instructions.

### RNA quantification

Total RNA, after lysis of cells in QIAzol lysis reagent (Qiagen), was purified using phenol-chloroform extraction. cDNA was synthetized using reverse transcriptase from Bio-Rad Laboratories. The amount of specific transcripts was measured by qRT-PCR using QuantiTect SYBR-Green PCR kit (Bio-Rad Laboratories) in the presence of specific primers synthesized by PRIMM (Table I) through the DNA Engine Opticon Real-Time PCR detection system (Bio-Rad Laboratories); each assay was run in triplicate. The relative amount of specific transcripts was calculated by the comparative cycle threshold method (27). The amount of GAPDH and β-actin transcripts was measured to normalize specific RNA levels.

The mRNA half-live was calculated by the equation (t_1/2_=Ln(0.5)/slope). The oligonucleotides used for DRB mRNA quantification were primers common to all DRB1 alleles (7).

### Chromatin immunoprecipitation (ChIP)

ChIP assay was performed on M14 and M14-p48 as previously described (7). The pre-cleared chromatin was immune-precipitated with 5 μg of anti-RNA Pol II CTD repeat P-S5 antibody and rabbit anti-IgG as control (Abcam). One tenth of the immune-precipitated DNA and input DNA were analysed by qRT-PCR, using DRA-c-F (ATTTTTCTGATTGGCCAAAGAGTAATT) and DRA-c-R (AAAAGAAAAGAGAATGTGGGGTGTAA) promoter primers.

### Western blot analysis

Western blot analysis was carried out using cell extract prepared using either RIPA buffer (10 mM Tris-HCl, pH 7.6, 150 mM NaCl, 1% NP-40) or PARIS kit (Ambion) in order to separate nuclear from cytoplasmic extracts.

Clones overexpressing p42 and p48 isoforms were screened using mouse monoclonal anti-FLAG (Sigma-Aldrich) and rabbit polyclonal anti-EBP1 antibodies (Millipore). Monoclonal anti-α-tubulin (Sigma-Aldrich) and anti-GAPDH (Abcam) were used to normalize cytoplasmic extract and anti-Laminin A+C (Abcam) for nuclear extract. STAT1 and pSTAT-1 were assessed by specific antibodies (BD Bioscience).

All membranes were developed using the ECL kit (Bio-Rad Laboratories) and exposed to X-ray film.

### Statistical analysis

All experimental results are the mean of three independent experiments. Statistical analysis was performed using the unpaired Student’s t-test with two-tailed distribution and two-sample equal variance parameter. In the figures, single asterisk corresponds to P<0.05 and double asterisk corresponds to P<0.01.

## Results

### Ectopic expression of EBP1 isoforms reveals a different phenotype in glioblastoma and melanoma vs. breast cancer

We performed a comparative analysis of the phenotype induced by the overexpression of p48 and p42 EBP1 isoforms in MCF7 breast carcinoma, U87 glioblastoma and M14 melanoma cell lines. Constructs FLAG-p48 and FLAG-p42 were used to obtain stable cells lines and the clones expressing ectopic FLAG proteins were selected by western blot analysis with anti-FLAG antibody. We confirmed comparable transfection efficiency by qRT-PCR measurement of FLAG mRNAs (data not shown).

**Figure 1:**
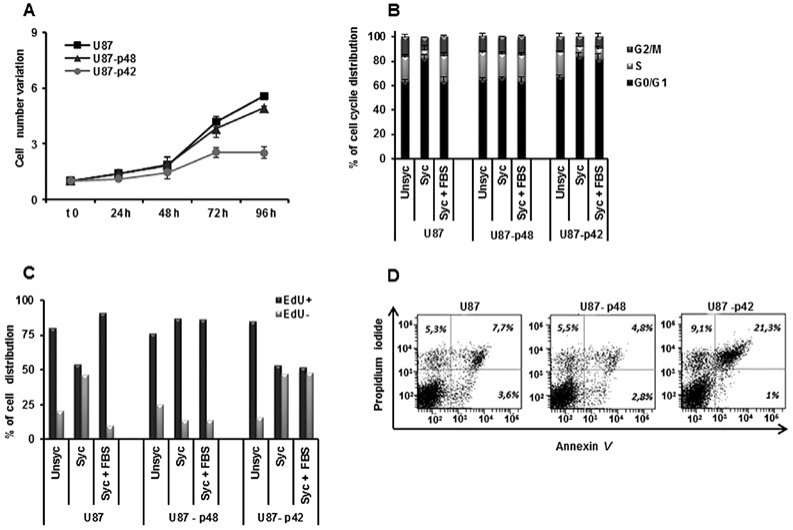
Proliferation phenotype of U87 glioblastoma cells overexpressing p42 and p48 EBP1 isoforms. (A) Growth rates evaluated at different time points (h). (B) Analysis of cell distribution in G2/M, S and G0/G1 phases of cell cycle. (C) Analysis of cell distribution following EdU incorporation. Cells were cultured in the presence of FBS (Unsyc), synchronized in the absence of serum (Syc) and released into the growth media containing FBS (Syc+FBS). (D) Quantitation of apoptotic cells by double staining with Annexin V and propidium iodide (PI). The dot plots show the percentage of cell distribution in early apoptosis (positive for Annexin V staining), late apoptosis (double positive for both Annexin V and PI staining) and necrotic cells (single positive for PI staining). A representative experiment for each transfectant is shown.

First of all, we evaluated the effect of p48 and p42 overexpression on the proliferation of the selected clones. We determined growth kinetics, by counting U87-p48 and U87-p42 cells and we observed that p42 slows down the cell growth, while p48 has no effect (Fig. 1A), consistent with published data (21). Examining the cell cycle progression of glioblastoma, we observed that synchronization does not block proliferation, because U87-p48 cells persist in S-phase also in the absence of serum (Fig. 1B). In contrast, p42 overexpression retains cells in G1 phase (81%) as compared to U87-p48 (63%) and to the control (65%) also during the restart of the cycle (Fig. 1B).

In order to endorse the differences in the dynamic proliferation between two stable cell lines, we performed EdU staining which incorporation occurs in the cycling cells. We observed that, during the synchronization phase, 85% of U87-p48 cells were EdU labelled. The percentage was unchanged in the subsequent 24 h of cycle restart, indicating that the cells remain in the S phase and continue to synthesize DNA (Fig. 1C). Otherwise, the number of U87-p42 cycling and not cycling are similar in synchronization and released phases, suggesting a block in the G1/S transition. The double staining with Annexin V and PI, 24 h after synchronization, clarified the consequences of the proliferative block induced by p42 and we observed 21.3% apoptotic U87-p42 cells (Fig. 1D), indicating that the block of G1/S transition induced apoptosis.

**Figure 2:**
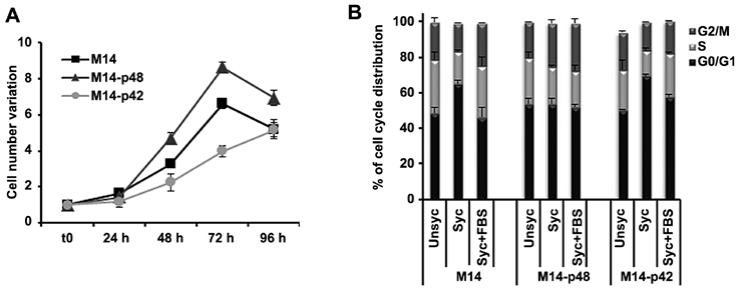
Proliferation phenotype of M14 melanoma cells overexpressing p42 and p48 EBP1 isoforms. (A) The cell growth rate of M14, M14-p42 and M14-p48 evaluated at different time-points. (B) Analysis of cell distribution of in G2/M, S and G0/G1 cell cycle phases.

In parallel, we analysed the phenotype of M14 melanoma tumors overexpressing p42 and p48. M14-p48 cells showed an increased cell growth rate (Fig. 2A), and a permanent distribution of cells in S phase of the cell cycle, also during synchronization (Fig. 2B) with a phenotype similar to U87-p48. Our data indicated that p48 isoform functions as oncogene in glioblastoma and melanoma by increasing cell proliferation, while p42 acts as a suppressor, by blocking proliferation and favouring apoptosis.

**Figure 3:**
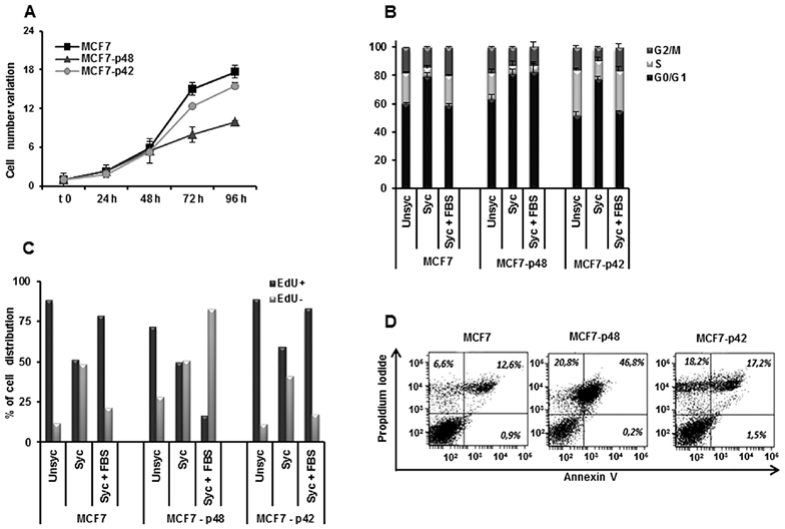
Proliferation phenotype of MCF7 breast carcinoma cells overexpressing p42 and p48 EBP1 isoforms. (A) Growth rates evaluated at different time-points (h). (B) Analysis of cells distribution in G2/M, S and G0/G1 phases of the cell cycle. (C) Analysis of cell distribution following EdU incorporation. (D) Quantitation of apoptotic cells by double staining with Annexin V and PI.

Otherwise, the overexpression of p48 in MCF7 cells leads to an inhibition of cell growth rate as compared to MCF7-p42 (Fig. 3A), in agreement with published data (11,28).

The analysis of the cell cycle (Fig. 3B) showed a significant increase of MCF7-p48 cell distribution in G1 phase (82%) as compared to MCF7 and MCF7-p42 cells (55 and 59%, respectively), associated with a drastic reduction of cells in S phase (4.7% in MCF7-p48 vs. 21% in MCF7 and 28% in MCF7-p42). EdU staining confirmed these differences and showed that, during the restart of the cycle, only 15% of MCF7-p48 is represented by proliferating cells as compared to 80% of MCF7-p42 (Fig. 3C). The block of MCF7-p48 proliferation determined the induction of apoptosis with 46.2% Annexin V-PI double positive (Fig. 3D) cells. In conclusion, p48 functions as tumor suppressor in breast carcinoma inducing apoptosis, while p42 does not affect the cell phenotype.

### EBP1 increases MHC class II expression

**Figure 4:**
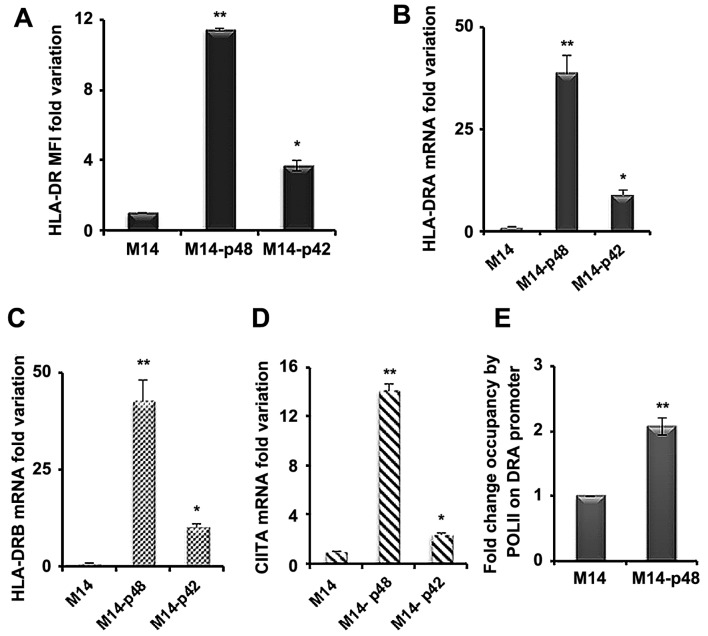
Analysis of MHC II expression in M14 transfectants. (A) Cytofluorimetric analysis of HLA-DR surface expression of p42 and p48 cells plotted as MFI (mean fluorescence intensity). Quantification of (B) DRA, (C) DRB and (D) CIITA mRNAs of transfected cells by qRT-PCR. mRNA amount is plotted as fold-change. p-value is relative to cells transfected by empty vector. (E) ChIP assay of M14 and M14-p48 cells is shown as HLA-DRA promoter occupancy by Pol II.

We have previously demonstrated that the knock-out of p48 EBP1 protein affects the expression of MHC II molecules (8). To further investigate the mechanism by which EBP1 influences MHC II regulation, we measured the HLA-DR surface level in M14 transfected cell lines. We showed that HLA-DR MFI is 10- to 12-fold higher in M14-p48 and 4-fold increased in M14-p42 cells compared to the control (Fig. 4A). To determine whether the surface increase of DR molecules is supported by an equal mRNA rise, we assessed the mRNA variations by qRT-PCR. We observed 40- to 50-fold increase in the amount of HLA-DRA and HLA-DRB mRNAs (Fig. 4B and C) in M14-p48 and 9-to 11-fold increase in M14-p42 cells. A significant increment was also observed for HLA-DQA1 mRNA in the M14-p48 cells, while MHC class I (MHC I) mRNA was unchanged (data not shown). In order to establish whether the variation of MHC II molecules was determined at the transcription level, we measured the expression of CIITA transactivator mRNAs and Pol II activity on DRA promoter. We observed a 14-fold increase of the total CIITA mRNA in M14-p48 cells compared to 3-fold rise in M14-p42 (Fig. 4D), indicating that MHC II over-expression is caused by a transcriptional control involving CIITA activation.

Moreover, it was confirmed that the CIITA increase corresponds to the higher Pol II activity by ChIP assay. We measured the endogenous promoter activity of DRA gene, encoding for alpha mRNA, and we observed that the fold occupancy of DRA promoter in M14-p48 cells by Pol II is double compared to M14 transfected with empty vector, demonstrating that p48 isoform affects MHC II transcription (Fig. 4E).

**Figure 5:**
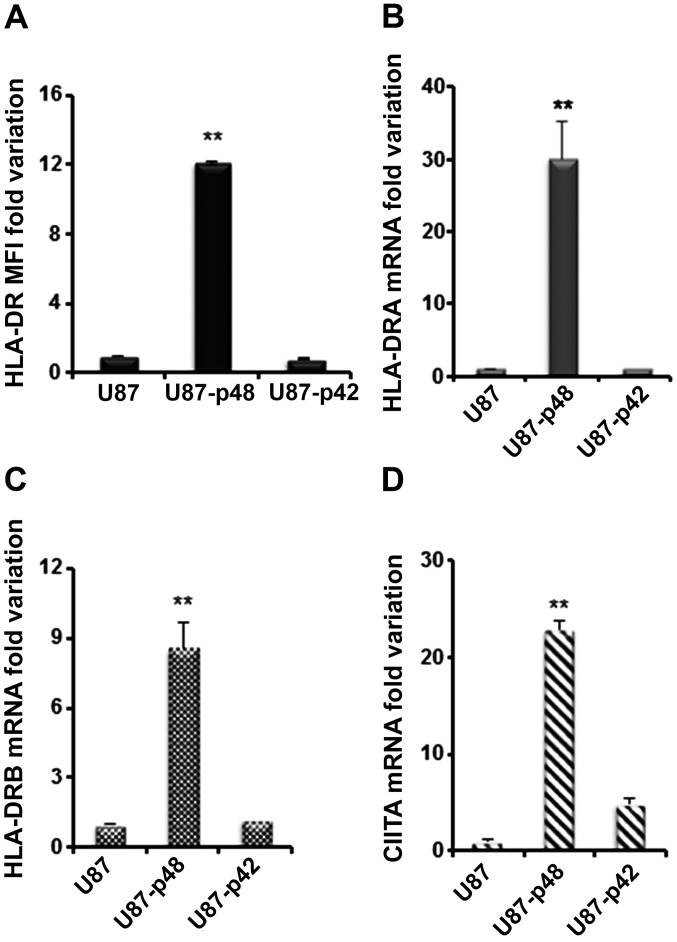
Analysis of MHC II expression in U87 transfectants. (A) Cytofluorimetric analysis of HLA-DR surface expression of p42 and p48 cells, plotted as MFI (mean fluorescence intensity). Quantification of (B) DRA, (C) DRB and (D) CIITA mRNAs by qRT-PCR of transfected cells.

We then analysed the effect of p42 and p48 isoforms overexpression on MHC II in glioblastoma. HLA-DR surface expression in U87-p48 showed a 12-fold increase as compared to U87-p42 and to control (Fig. 5A). The surface variation corresponds to 30-fold increase of DRA mRNA (Fig. 5B) and 9-fold increase of DRB mRNA (Fig. 5C) in U87-p48 cells, while no variation was observed in the case of U87-p42. The measurement of DQA1 and MHC I mRNAs (data not shown) confirmed data obtained in M14. In addition, we quantified the CIITA mRNA amount and we found 23-fold increase in U87-p48 and 5-fold increase in U87-p42 (Fig. 5D). Similarly to the results obtained with M14, we demonstrated that in glioblastoma cell line p48 protein increases the transcription of MHC II genes through CIITA activation.

**Figure 6:**
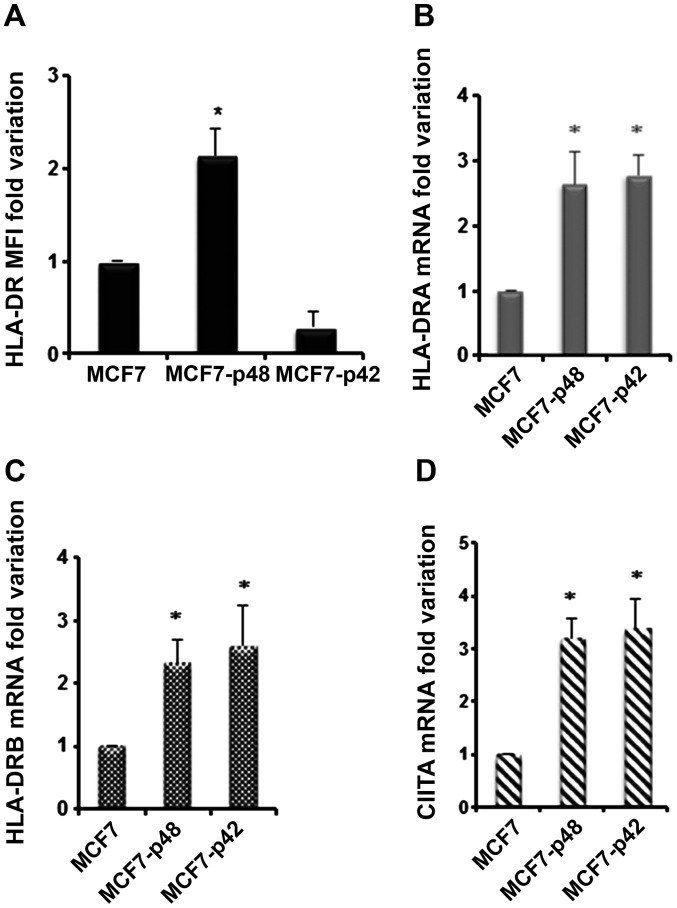
Analysis of MHC II expression in MCF7 transfectants. (A) Cytofluorimetric analysis of HLA-DR surface expression of p42 and p48 cells, plotted as MFI (mean fluorescence intensity) quantification of (B) DRA, (C) DRB and (D) CIITA mRNAs by qRT-PCR of transfected cells.

Finally, we used MCF7 cells which express MHC II molecules only upon stimulation with IFNγ. Since we performed the same treatment in the control, we assumed that any variation should be attributed to the difference between the two stable cell lines. We determined HLA-DR surface expression of MCF7 transfected cell lines after IFNγ. As shown in Fig. 6A, we observed that HLA-DR MFI of MCF7-p48 shows 2.2-fold increase compared to the cells transfected with empty vector, while MCF7-p42 showed a downregulation of HLA-DR expression as compared to the control. Surprisingly, we observed a 4-fold increase in the amount of HLA-DRA and HLA-DRB not only in p48 but also in p42 transfectants (Fig. 6B and C), which does not correspond with an increase in MHC II protein (Fig. 6A). The expression of DQA1 is comparable to DR mRNAs while MHC I mRNA is unchanged (data not shown). In order to explain the MHC II mRNA differences, we analysed CIITA mRNA that showed 3-fold increase in both MCF7-p48 and MCF7-p42 (Fig. 6D). In conclusion, both isoforms in carcinoma determine a comparable CIITA activation and MHC II transcription, with only p42 isoform being able to block MHC II protein synthesis through a mechanism which is currently under investigation.

### p48 oncogene influences the STAT-1 pathway

**Figure 7:**
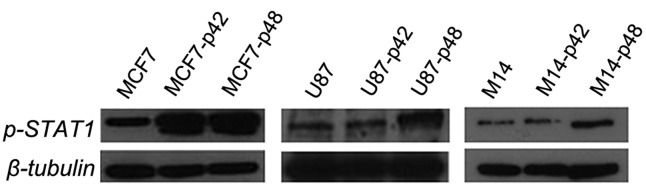
Analysis of STAT1 phosphorylation and mRNA decay in U87, MCF7 and M14 transfectants. Western blot analysis of MCF7, U87 and M14 cell extracts overexpressing p42 and p48 isoforms performed by antip-STAT1. Anti-β-tubulin was used to normalize the amount of proteins.

In order to unravel the protein pathway activation upstream of CIITA, we evaluated the STAT1 phosphorylation in all cell lines overexpressing EBP1 isoforms. We observed that both U87 and M14 cells, constitutively expressing MHC II, show upregulation of p-STAT1 in the p48 overexpressing cells only and not in the p42 cells (Fig. 7). These results clearly demonstrate that p48 induces STAT1 phosphorylation in the absence of cytokine stimulation when it acts as an oncogene in U87 and M14. For MCF7-p42 and MCF7-p48 cells, we observed no differences in the phosphorylation status of STAT1, and this in agreement with the comparable CIITA increase (Fig. 6D).

### p48 isoform regulates MHC II stability

**Figure 8:**
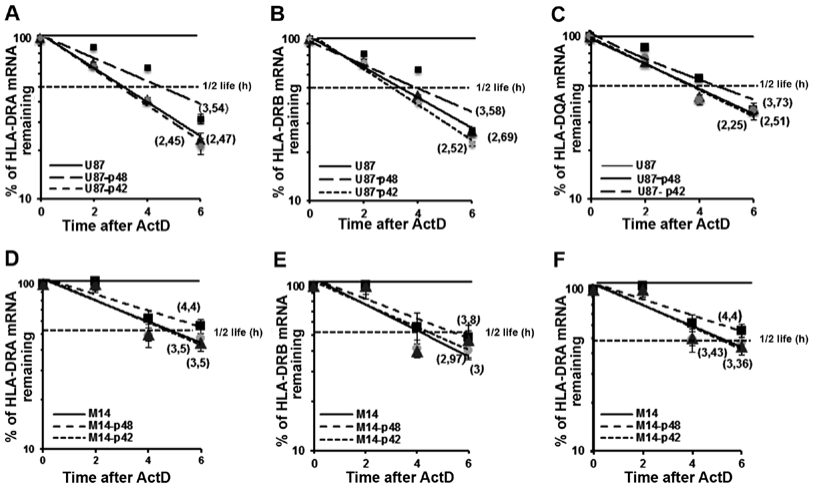
Kinetics of mRNA decay in U87 and M14 transfected cells. Analysis of (A and D) DRA, (B and E) DRB and (C and F) DQA1 mRNAs remaining following 2, 4 and 6 h of ActD, are shown as percentage on y-axis, in U87 and M14 transfected cells. The half-life of mRNAs, shown in parentheses, represents the required time for a given transcript to decrease to 50% of its initial abundance.

We already demonstrated that p48 isoform influences MHC II posttranscriptional regulation through the binding to UTRs of the messengers (7,8). In the present study, we evaluated the kinetics of mRNA decay in U87-p48 and U87-p42 cells after blocking transcription at 2, 4 and 6 h with ActD. Total mRNA was analysed for DRA, DRB and DQA1 expression by qRT-PCR, normalized by β-actin mRNA level and expressed as percentage of maximum. When calculating the half-lives of endogenous mRNAs in glioblastoma cells transfected with p48, we found that DRA mRNA (t_1/2_=3.54 h; Fig. 8A) was more stable than the DRA messenger from cells transfected with p42 or empty vector (t_1/2_=2,45 h; Fig. 8A). Also, the steady-state of DRB (Fig. 8B) and DQA1 (Fig. 8C) mRNA in cells overexpressing p48 resulted in an increase of almost 1 h as compared to mRNAs from U87-p42 cells. This increase was also demonstrated in M14-p48 cells as shown in Fig. 8D–F. In conclusion, the overexpression of p48 isoform, through the binding to 3′UTR of transcripts, decreases endogenous MHC II mRNA decay, while p42 isoform is not able to interact with UTRs and influence their stability.

## Discussion

Recent studies have shown that CD4^+^ T cells are critical for the generation and persistence of CTL responses by providing help through multiple interactions with antigen-presenting cells or by directly providing co-stimulatory signals to the CTLs, which enhance their function and survival at the tumour site. In addition, CD4^+^ T helper cells may function as effector cells either by the local production of cytokines (IFNγ and IL2) that curtail tumour growth or trigger the release of cytotoxic mediators towards MHC II tumour cells (29,30).

All these findings emphasize the interest for the MHC II expression by tumour cells, which is variable and not clearly related to the clinical outcome (31). Although the majority of solid cancers do not constitutively express MHC II at significant levels and cannot be directly recognized or killed *in vitro* by CD4^+^ lymphocytes, many tumour cells can upregulate the MHC II upon stimulation with IFNγ. Cytokine stimulation activates CIITA expression as consequence of promoter IV epigenetic de-repression (32). Moreover, no data are available to correlate the function of specific oncogenes with MHC II activation in solid tumours.

We have previously demonstrated that MHC II mRNAs are regulated at post-transcriptional level by an RNP complex that affects the processing and guarantees a coordinate expression of mRNAs encoding two chains of MHC heterodimeric molecules. One of the factors involved in the RNP complex is p48 isoform of EBP1, an RNA binding protein that, interacting with UTRs of MHC II messengers, affects MHC II post-transcriptional regulation (7,8).

In the present study, we first studied the effect of the p48 and p42 EBP1 isoforms on cell cycle in different tumour cell lines of non-hematopoietic origin, and then analysed the role of EBP1 isoforms on MHC II expression.

Many studies reported that p48 isoform represses transcription of genes involved in cell cycle progression in the nucleus of carcinoma, with consequent inhibition of proliferation (10–12,15,28,33). In these papers, it has been showed that p48 was able to interact with retinoblastoma (Rb) protein through the binding of the C-terminal region to form a repressor complex with Sin3A and HDAC2, which tightly binds E2F family proteins preventing the transcription of E2F regulated cell cycle genes (14,34,35). Others authors have demonstrated that in glioblastoma p48 isoform induces proliferation, *in vitro* and *in vivo*, because it causes p53 poly-ubiquitination and degradation trough the interaction with HDM2, while p42 reduces growth and promotes differentiation (20,21,36).

In the present study, we have analysed the different role of the two isoforms in tumorigenesis using three different cell lines: glioblastoma (U87), melanoma (M14) and a breast carcinoma (MCF7), overexpressing p48 or p42. We assessed in parallel the effect of p48 and p42 on cell cycle and apoptosis, in relationship with the cell type. In MCF7-p48, we observed a block of cell proliferation and a strong induction of apoptosis, whereas, the overexpression of p42, does not show differences in the cell cycle progression as compared to the control. In this case, we confirmed the anti-proliferative and apoptotic functions of p48, already demonstrated in different types of carcinoma, such as breast, prostate, bladder and hepatocellular carcinoma (11,18,19,28). In contrast, we found that, in glioblastoma and melanoma, p48 overexpression increases proliferation by blocking cells in S phase, thus confirming the phenotype already observed (36–38). Furthermore, we demonstrated for the first time that p42 isoform inhibits proliferation of glioblastoma, by blocking cells in G1 phase of the cell cycle and by inducing apoptosis. In conclusion, our findings confirm that p48 acts as an oncogene in glioblastoma and as an onco-suppressor in carcinoma.

We then performed the analysis of MHC II expression pattern in cells overexpressing p48 and p42 and we found that the overexpression of p48 increases the surface amount of HLA-DR heterodimer in U87, MCF7 and M14 cell lines and this phenotype is due to increased transcription and mRNA stability of DRA and DRB genes. MHC II transcriptional activation is a consequence of the CIITA transactivator activity, that occurs in normal and tumour cells of hematopoietic origin, as well as in several tumours of nonhematopoietic origin and in a consistent number of cell lines (e.g., glioblastoma, pancreas adenocarcinoma, melanoma and bladder carcinoma, ATLAS data base), in some cases after IFNγ stimulation. In this study, the cell lines overexpressing p48 show a strong increase of CIITA in M14 and U87, and a lower but significant three-fold increase in MCF7, following IFNγ activation. Next, we investigated the pathway upstream of CIITA transactivator, especially the STAT1 protein, that activates CIITA transcription through the binding to GAS (interferon-gamma-activated sequence) element in the CIITA promoter, upon cytokine stimulation (5). We found higher level of STAT1 phosphorylation in both U87-p48 and M14-p48 cells indicating a clear involvement of this transcription factor in the CIITA and MHC II upregulation through p48 protein. U87 and M14 overexpressing p42 isoform did not show any variation of MHC II, CIITA or p-STAT1 protein levels with respect to the control. On the other hand, MCF7 shows a comparable level of STAT1 phosphorylation in p48 and p42 overexpressing cells, that explains the increase of CIITA mRNA but not the difference in HLA-DR surface protein. These results probably could be dependent on post-transcriptional regulation which is currently under investigation.

In our previous study, we showed that p48 interacts with UTRs of MHC class II mRNAs (8), here we further investigated its role during RNA processing. We show a clear increase of DRA, DRB and DQA1 mRNA half-lives in U87-p48 and M14-p48. No variation of mRNA decay was observed in cells overexpressing p42 probably because this isoform lacks of a specific RNA binding motif at the N-terminus of the protein (39).

In conclusion, the present study explored the different roles of two EBP1 isoforms, analysing them simultaneously in overexpressing cell lines and confirming their influence on the tumour phenotype in a cell-type specific manner. p48 showed an anti-proliferative and apoptotic role in carcinoma versus a proliferative function in glioblastoma and melanoma. In this regard, we hypothesize that in glioblastoma the different function of p48 may be regulated by HDM2 that is able to interact with both tumour suppressors p53 and Rb (40). Conversely, p42 isoform shows a clear function of tumour suppressor, inhibiting proliferation and inducing apoptosis.

One hallmark of tumour cells is the acquisition of a new profile of expressed proteins, determined by changes in gene regulation. In this contest, the ability of tumour cells to express MHC II could be a consequence of p48 EBP1 activity that upregulates MHC II expression at transcriptional level via STAT1 activation and by a post-transcriptional regulation, which increases mRNAs stability.

We propose a scenario in which, when an oncogene such as p48 affects the normal gene expression and induces tumor progression, the increment of MHC II surface molecules can be considered a strategy used by cells to activate a tumor-specific immune response of CD4^+^ T cells.

## Figures and Tables

**Table I:** Primers used for qRT-PCR.

Gene	Primers	Sequences 5′→3′	Annealing temperature (°C)	Fragment size
β-actin	ACT-F	TCATGAAGTGTGACGTTGACA	58	285 nt
	ACT-R	CCTAGAAGCATTTGCGGTGCAC		
GAPDH	G-F	AACGGATTTGGTCGTATTGGC	58	216 bp
	G-R	TCGCTCCTGGAAGATGGTGATG		
HLA-DRA	DRA-F	GGACAAAGCCAACCTGGAAA	60	120 bp
	DRA-R	AGGACGTTGGCTCTCTCAG		
HLA-DRB	DRB1-F	CTCAGCATCTTGCTCTTGTGCAG	60	228 bp
	DRB1-R	CAGCATTAAAGTCAGGTGGTTCC		
HLA-DQA1	DQA1-F	GTGTAAACTTGTACCAGT	58	263 bp
	DQA1-R	GAGACTTGGAAAACACT		
HLA-A,B,C	MHCI-F	AGTGGGCTACGTGGACGACA	58	300 bp
	MHCI-R	ATGTAATCCTTGCCGTCGTA		
CIITA	CIITA-F	CCGACACAGACACCATCAAC	58	222 bp
	CIITA-R	CTTTTCTGCCCAACTTCTGC		
